# Supplementary material for: Phenotypic and metabolic plasticity shapes life‐history strategies under combinations of abiotic stresses
Source: Plant Direct. 2019 Jan 10;3(1):e00113. doi: 10.1002/pld3.113 (PMC6508786; doi:10.1002/pld3.113)
Supplement: Supplementary file 1 [file PLD3-3-e00113-s001.pdf]

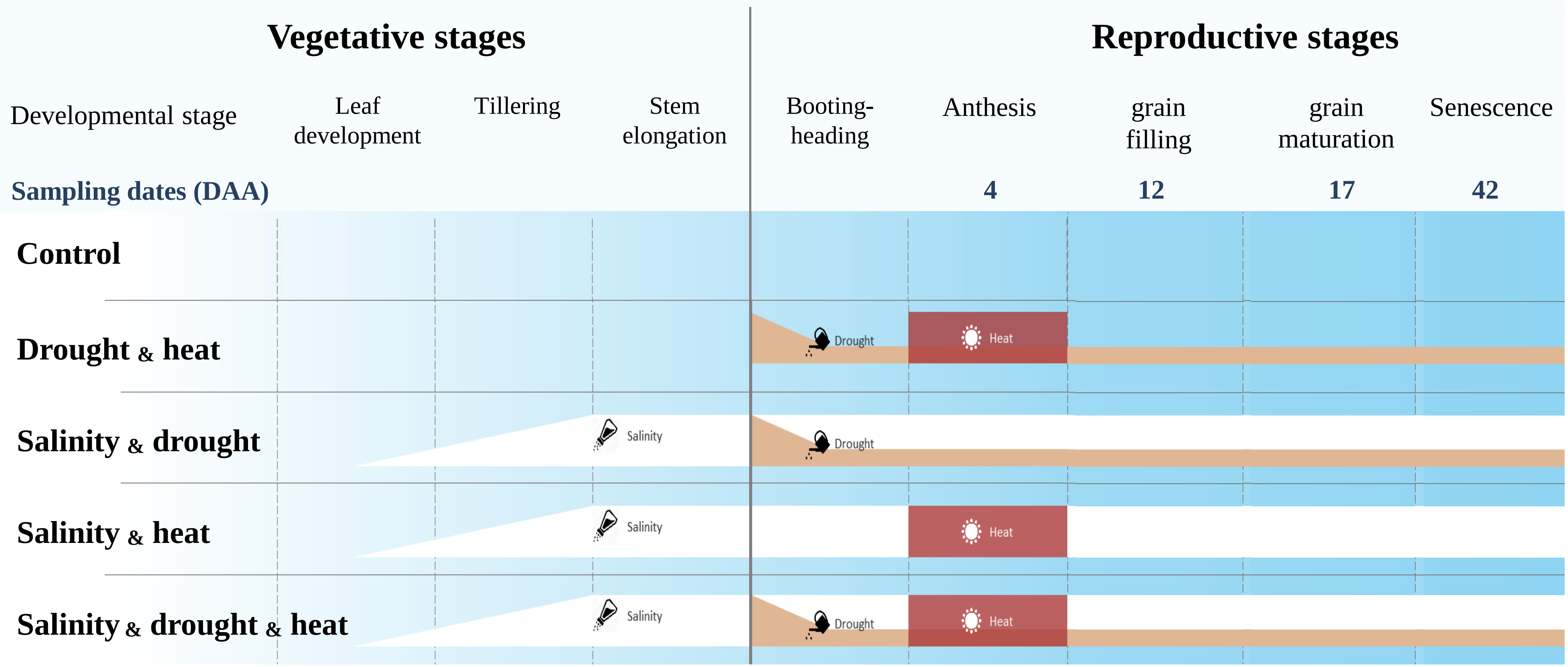

Figure S1. A schematic overview of the stress combination assay. *Brachypodium distachyon* plants were subjected to the stresses based on their developmental stages under the Mediterranean-like conditions (i.e. salinity stress was applied at leaf development, drought at booting-heading stages and heat at anthesis). Plants were samples at 4, 12, 17 and 42 days after anthesis (DAA).
