# Supplementary material for: Phenotypic and metabolic plasticity shapes life‐history strategies under combinations of abiotic stresses
Source: Plant Direct. 2019 Jan 10;3(1):e00113. doi: 10.1002/pld3.113 (PMC6508786; doi:10.1002/pld3.113)
Supplement: Supplementary file 2 [file PLD3-3-e00113-s002.pdf]

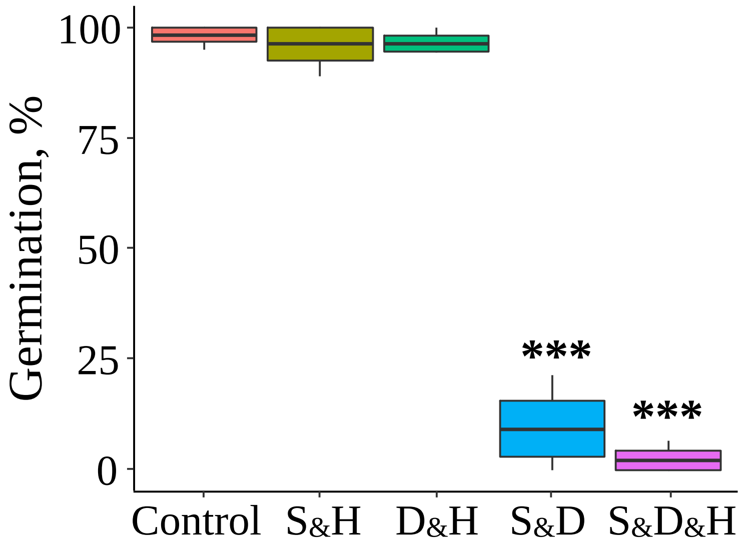

Figure S2. Box plot of germination rate of grains that developed on plants subjected to combinations of stresses. *** indicates a significant difference at $P < 0.001$, as determined by Dunnet's test. Values are mean (n=3) ± SE. Growth conditions are as follows: salinity and heat ($S_{\&}H$), drought and heat ($D_{\&}H$), salinity and drought ($S_{\&}D$), salinity, drought and heat ($S_{\&}D_{\&}H$).
