# Supplementary material for: Phenotypic and metabolic plasticity shapes life‐history strategies under combinations of abiotic stresses
Source: Plant Direct. 2019 Jan 10;3(1):e00113. doi: 10.1002/pld3.113 (PMC6508786; doi:10.1002/pld3.113)
Supplement: Supplementary file 3 [file PLD3-3-e00113-s003.pdf]

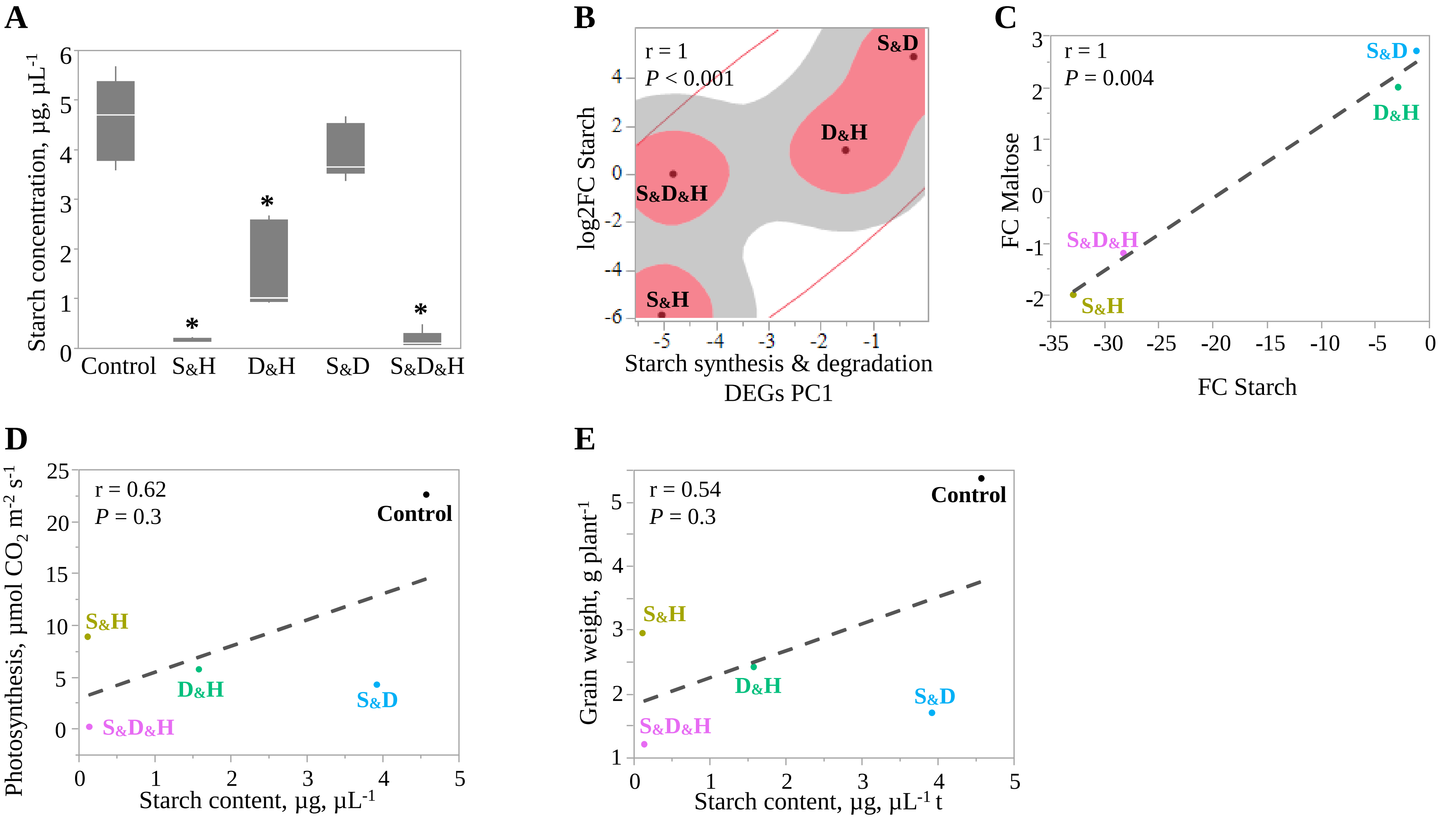

Figure S3. Starch content in leaves of *Brachypodium distachyon* and its association with physiological traits or gene expression pattern. (A) Box plot of starch content under control and combinations of stresses. * indicates a significant difference at $P \leq 0.05$, as determined by Steel method for nonparametric comparisons. Values are mean (n=5)± SE. (B) Correlation between first principle component (PC1) of expression pattern of genes involved in starch synthesis and degradation, and log2 fold change (FC) values of starch content. Correlations between starch content and (C) maltose, (D) photosynthesis and (E) grain weight. Growth conditions are as follows: salinity and heat ($S_{\&}H$), drought and heat ($D_{\&}H$), salinity and drought ($S_{\&}D$), salinity, drought and heat ($S_{\&}D_{\&}H$).
