# Supplementary material for: Phenotypic and metabolic plasticity shapes life‐history strategies under combinations of abiotic stresses
Source: Plant Direct. 2019 Jan 10;3(1):e00113. doi: 10.1002/pld3.113 (PMC6508786; doi:10.1002/pld3.113)
Supplement: Supplementary file 4 [file PLD3-3-e00113-s004.pdf]

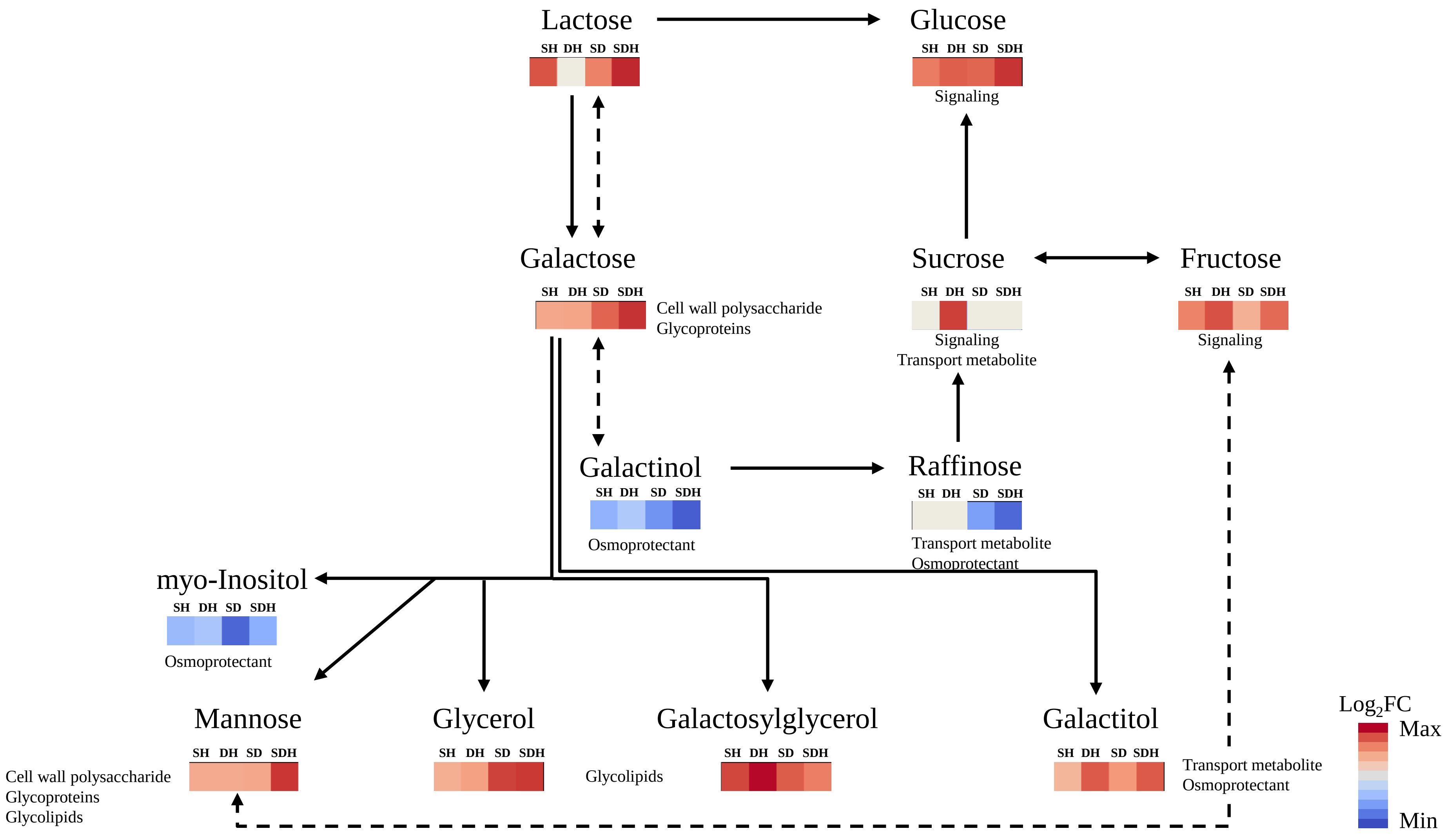

Figure S4. Galactose metabolic pathway under combinations of stresses. Metabolites of the galactose pathway that showed significant alterations in at least one stress combination ($P \leq 0.05$), compared with control. Red and blue colors indicate accumulation and depletion of metabolite content, respectively, and white color indicates a non-significant change, compared with control. Solid line denotes one enzymatic reaction and dashed line denotes involvement of several enzymatic reactions between two metabolites. Growth conditions are as follows: salinity and heat ($S_{\&}H$), drought and heat ($D_{\&}H$), salinity and drought ($S_{\&}D$), salinity, drought and heat ($S_{\&}D_{\&}H$).
