# Supplementary material for: Phenotypic and metabolic plasticity shapes life‐history strategies under combinations of abiotic stresses
Source: Plant Direct. 2019 Jan 10;3(1):e00113. doi: 10.1002/pld3.113 (PMC6508786; doi:10.1002/pld3.113)
Supplement: Supplementary file 5 [file PLD3-3-e00113-s005.pdf]

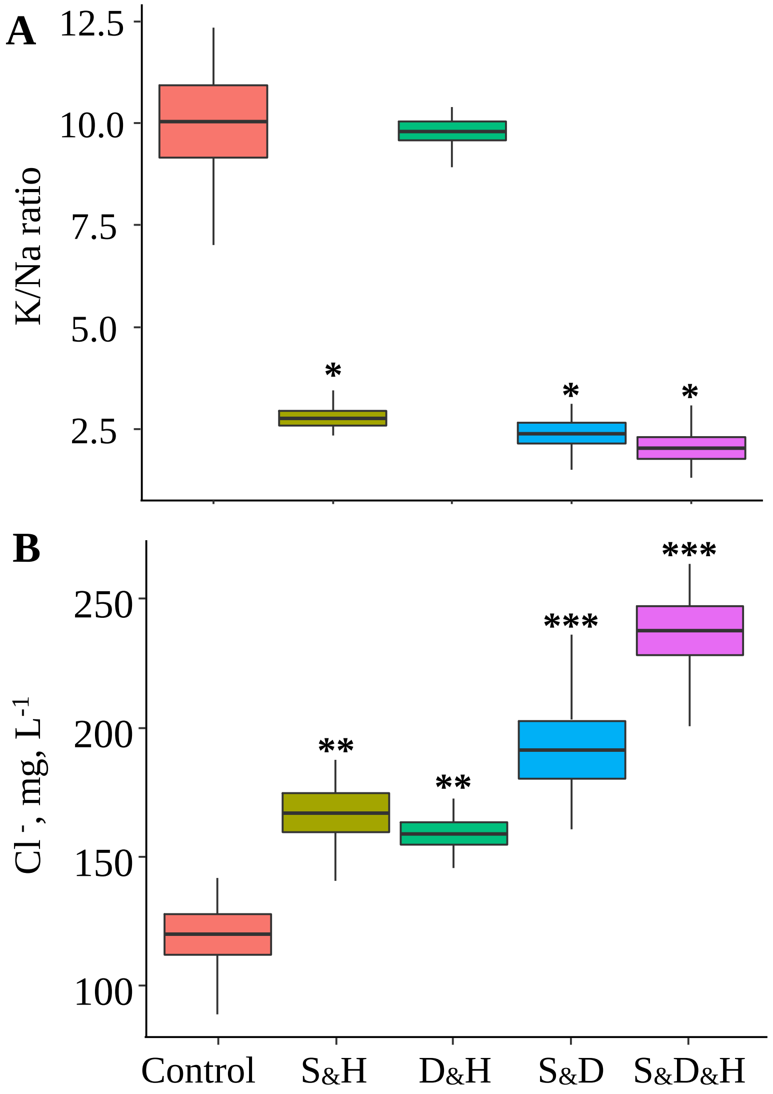

Figure S5. Potassium / Sodium ratio and chloride concentration in *Brachypodium distachyon* leaves under control and combinations of stresses. Box plots of (A)the ratio between Na^+ and K^+ and (B) Cl^-. *, **, and *** indicate a significant difference at $P \leq 0.05$, 0.01, and 0.001, respectively, as determined by Dunnet's test. Values are mean (n=6) ± SE. Growth conditions are as follows: salinity and heat ($S_{\&}H$), drought and heat ($D_{\&}H$), salinity and drought ($S_{\&}D$), salinity, drought and heat ($S_{\&}D_{\&}H$).
