# Supplementary material for: Phenotypic and metabolic plasticity shapes life‐history strategies under combinations of abiotic stresses
Source: Plant Direct. 2019 Jan 10;3(1):e00113. doi: 10.1002/pld3.113 (PMC6508786; doi:10.1002/pld3.113)
Supplement: Supplementary file 7 [file PLD3-3-e00113-s007.pdf]

האוניברסיטה העברית בירושלים
The Hebrew University of Jerusalem
המכון למדעי הצמח וגנטיקה בחקלאות ע"ש רוברט ה. סמית
The Robert H. Smith Institute of Plant Sciences and Genetics in Agriculture
Prof. Zvi Peleg פרופ' צבי פלג

11-December-2018

Dear Editor,
Prof. David Des Marais

Re: Revision of "***Phenotypic and metabolic plasticity shapes life-history strategies under combinations of abiotic stresses***" (Plant Direct: 2018-00217).

Thank you for your letter dated November 16, 2018, notifying us that our manuscript may be acceptable for publication in *Plant Direct* following revision.

The manuscript was revised according to the comments made by the two reviewers (*in Italics*) as specified below.

<u>In response to the comments of reviewer #1:</u>

Pg. 4 Lines 1-2. Please provide a citation for that statement.

- We have provided the statement with a relevant citation as suggested.

Pg. 9 Line 14. "linear" instead of "liner"

- We thank the reviewer for noticing. The typo has been corrected.

<u>In response to the comments of reviewer #2:</u>

Page 5 line 11, what is n for each treatment? Treatments were applied at different ages, can the authors provide a clear justification for this in the methods?

- We thank the reviewer for this comment. The number of biological replicates for each trait is denoted in the methods section, and varied between three to six replicates, depending on the trait. Transcriptional responses were evaluated based on three biological replicates, and metabolic profiles and ion concentrations were assessed based on six biological replicates.
- We applied each stress at specific developmental stage in an attempt to mimic the natural development of stresses under field conditions. Salinity stress was gradually applied starting at early developmental stage (i.e. five leaf-stage), to mimic the progressive development of salinity stress during the growing season. Drought was applied gradually by withholding water at booting stage to simulate the reduction of soil water availability, as the plants transition to the reproductive stage during the Mediterranean spring, which is characterize by rain ceasing. Finally, heat stress was applied at anthesis for four days similarly to a typical Mediterranean spring heatwave. We revised the methods accordingly, as suggested.

Page 7 line 29, which leaf samples were these? In other sections the type of leaf material is specified.

- We have added the required details on the type of leaf samples that were used to determine starch content.

Page 8 line 2, specify which soluble sugars were measured.

- We have revised the text, as suggested, and replaced the phrase “soluble sugars” with “glucose”.

Page 9 line 23, the authors have defined reproductive allocation as %; however, the absolute values of reproductive parameters do decrease in S&H plants. Suggest revising the subheading to better reflect these data.

- We thank the reviewer for this valuable comment. The impairment of plant performances and specifically of yield parameters under combined stresses is emphasized in the text (page 10, line 25- page 11, line 31). Indeed, under S&H, yield parameters were decreased, yet the proportion of spike dry weight per shoot dry weight, which was used as a surrogate for reproductive allocation, was comparable to control conditions. The similar allocation of the limiting resources was unique to the combination of S&H and was not detected among the other stress combinations. As suggested, we revised the subheading to better reflect this result.

Figures 1, 2, 4 the axis text is unclear.

- We have submitted high resolution Figures with the revised MS revision.

Figure 2A the error bars do not align with the trend lines.

- We have used an exponential model to best fit the data, thus, the bars do not align with the line.

Finally, we wish to thank you for handling the revision of our manuscript and the reviewers for their helpful comments. We truly believe that the current version of the manuscript is considerably improved and hope that you will find it suitable for publication in *Plant Direct*.

Yours sincerely,

Z. Peleg

Prof. Zvi Peleg
Faculty of Agriculture, Food and Environment
The Hebrew University of Jerusalem
Tel: +972-8-9489-638; Fax: +972-8-9489833
E-mail: zvi.peleg@mail.huji.ac.il
